# External cephalic version for a malpresenting first twin before labor: a prospective case series

**DOI:** 10.1016/j.xagr.2022.100122

**Published:** 2022-10-15

**Authors:** Laura Felder, Rebekah McCurdy, Vincenzo Berghella

**Affiliations:** Division of Maternal-Fetal Medicine, Department of Obstetrics and Gynecology, Sidney Kimmel Medical College, Thomas Jefferson University, Philadelphia, PA

**Keywords:** external cephalic version, malpresenting twin pregnancy, twin delivery, twin pregnancy

## Abstract

**BACKGROUND:**

In twin pregnancies where the presenting twin is not cephalic, cesarean delivery is the standard of care. External cephalic version (ECV) has been used for malpresenting singleton pregnancies with low risk of complications. ECV in twin pregnancies is poorly studied.

**OBJECTIVE:**

To assess feasibility and report any complications of ECV of a malpresenting twin before labor.

**STUDY DESIGN:**

This is a prospective cohort of twin pregnancies with malpresenting first twin. Inclusion criteria included English or Spanish speaking women. Exclusions included cases where there was a contraindication to vaginal delivery. ECV was performed according to the institutional singleton protocol. Fetal testing of both twins was performed before and after procedure. A vaginal hand was used during ECV as needed. The primary outcome was success of the procedure. Secondary outcomes included delivery characteristics and neonatal outcomes.

**RESULTS:**

Five patients were enrolled in this study. Four patients underwent successful ECV and vaginal delivery occurred in 2 of the 4 patients. ECV procedure was performed at a mean gestational age of 36+0 weeks in the successful ECV group and 36+6/7 weeks for the unsuccessful group. Latency to delivery was 4.5 days in the successful ECV group and 1 day in the unsuccessful ECV group. No maternal or neonatal complications occurred in any participating women.

**CONCLUSION:**

ECV in twin pregnancies where the first twin is malpresenting was feasible in our cohort. More research is needed to better characterizer the safety and efficacy of this procedure in this patient population.

**AJOG Global Reports at a Glance**

**Why was this study conducted?**

The study was conducted to determine the feasibility of external cephalic version in malpresenting twin pregnancies.

**Key findings**

External cephalic version was successful in 4 of 5 malpresenting twin pregnancies. No complications occurred in our study participants.

**What does this add to what is known?**

External cephalic version is feasible in malpresenting twin pregnancies.

## Introduction

Twin gestations are increasing in prevalence and are associated with a high cesarean delivery (CD) rate relative to that of singleton pregnancies. CD carries a number of maternal and fetal risks compared with vaginal delivery, even in twins. The Twin Birth Study failed to show an improvement in neonatal outcomes when planned CD was performed for multiple gestations in which the first twin was cephalic.^1^

External cephalic version (ECV) is a procedure performed in singleton pregnancies complicated by malpresentation to avoid CD; the standard of care for malpresentation is otherwise CD. ECV for singletons is successful approximately 60% of the time, and complications occur in <1% of cases.^2^^,^^3^ ECV for twins with malpresentation of the first twin is uncommonly performed but has been previously described.^4^^,^^5^

We offered ECV to patients with twin gestations and malpresentation of the first twin who strongly desired vaginal delivery, and aimed to assess the feasibility of the procedure and capture any adverse outcomes.

## Materials and Methods

Institutional review board approval was obtained for this study. Patients with diamniotic twin pregnancies in which the first twin had malpresentation in the third trimester were approached for participation. Patients were eligible if they were English- or Spanish-speaking, regardless of age or parity. Both dichorionic-diamniotic and monochorionic-diamniotic pregnancies were eligible for inclusion. Patients with contraindications to vaginal delivery were not eligible to participate. Consenting patients underwent ECV of the first twin within 2 weeks of their scheduled induction date on labor and delivery. ECV was performed in accordance with the institutional singleton ECV protocol. Patients were instructed to fast overnight. An intravenous line was placed on arrival, and spinal anesthesia and a uterine relaxant were recommended. Routine laboratory tests were collected and fetal monitoring of both infants was conducted by nonstress testing (NST). ECV of the first twin was performed by an attending physician with additional resident and fellow physicians. Bedside ultrasound was performed before the procedure and throughout. Hands on the maternal abdomen were used to turn the presenting twin. A vaginal hand was used as needed. After the procedure, approximately 2 hours of fetal monitoring with NST was conducted if discharge was planned.

The primary outcome was procedure success, defined as the presenting twin in cephalic presentation at the end of the procedure. Secondary outcomes included delivery characteristics and neonatal outcomes.

## Results

During the study period (October 2019–June 2022), 5 patients consented to participation (Table). Data on these patients have not been previously published. Their ages ranged from 24 to 34 years, and body mass index ranged from 29 to 40. All included twins were dichorionic-diamniotic, and gestational age ranged from 34+2/7 to 37+1/7 weeks. All patients had ≥1 previous deliveries. Two cases were complicated by fetal growth restriction, and 1 patient had no previous spontaneous vaginal deliveries (SVDs). All had normal fluid. Placental location and the position of the second twin varied.

**Table:** Maternal and neonatal outcomes

Outcomes	Successful ECV (N=4)	Unsuccessful ECV (N=1)
Gestational age (wk+d)	36+0 (34+2/7–37+1/7)	36+6/7
Twin A vertex immediately after ECV	4/4	0/1
Interval to delivery (d)	4.5 (0–16)^a^	1
Delivery route	SVD: 2/4 CD: 2/4	CD: 1/1
Twin A vertex at time of delivery	3/4	0/1
ECV complications (within 24 h of ECV)^b^	0/4	0/1
Twin A delivery weight (g)	2350 (1730–2750)	3459
Twin B delivery weight (g)	2328 (1460–2930)	3140
Twin A APGARS 1/5 min	8/9 (8–8)	8/8
Twin B APGARS 1/5 min	8 (5–9)	9/8
Readmission or ED visit for abdominal pain following ECV attempt	0/4	0/1

Data presented as number (range) or mean (range).

*APGARS*, Apgar score; *CD*, cesarean delivery; *ECV*, external cephalic version; *ED*, emergency department; *SVD*, spontaneous vaginal delivery.

a Labor induction planned for earlier date but delayed because of patient request

b Complications defined as rupture of membranes, cord prolapse, emergent CD, vaginal bleeding, nonreassuring fetal heart monitoring requiring admission/prolonged monitoring, fetal demise.

Four of 5 patients underwent successful ECV of the presenting twin. Two patients had SVD of both twins. CD was performed at complete dilation, before pushing, for 1 patient on maternal request. This patient had never had an SVD. CD was also performed in 1 patient for unstable lie after labor induction was initiated. All patients received regional anesthesia and a dose of uterine relaxant. The length of procedure ranged from 5 to 22 minutes. A vaginal hand was used for 2 patients. No adverse events occurred in any of the 5 participants.

## Discussion

ECV in dichorionic-diamniotic twin pregnancies complicated by malpresentation was successful in 4 of the 5 patients, all of whom were multigravidas. However, CD was still quite common, occurring in 2 of 4 successful ECVs. A recently published cohort of 23 patients undergoing ECV in twin pregnancy showed a success rate of 56%.^5^ No adverse events occurred in any of our patients. The technique for twin ECV did not differ significantly from that used in singleton ECV. Providers noted that when the presenting twin was anterior to the second twin, the procedure was perceived to be easier. All successful procedures occurred in fetuses with birthweight <3000 g. Ultimately, this study is limited by the very small number of participants but demonstrates feasibility for larger randomized controlled trials that may investigate the rate of vaginal delivery when CD is otherwise planned.
